# Use of Vastus Medialis Rotational Muscle Flaps to Cover Infected and Exposed Reverse Saphenous Vein Graft Following Traumatic Femoral Artery Injury

**DOI:** 10.7759/cureus.99755

**Published:** 2025-12-21

**Authors:** Samitha Senevirathne, Jayamini Kaushalya, Joel Arudchelvam, Liyanage A Indunil, Suren P Ruwan Pathirana

**Affiliations:** 1 University Vascular Surgical Unit, National Hospital of Sri Lanka, Colombo, LKA

**Keywords:** lower extremity vascular injury, open fractures, rotational muscle flaps, vascular graft exposure, vascular graft infection

## Abstract

Use of autologous and prosthetic grafts is one of the key concepts in open vascular reconstruction surgery. Despite taking different measures, there is still a chance of vascular graft infections due to various patient and surgery-related factors. Some of the vascular grafts might lose the overlying soft tissue cover, leading to graft exposure. Both these complications pose a significant threat to the graft viability. A graft with threatened viability poses a risk to the supplying end organ, limb, or life. Therefore, vascular graft exposure and graft infections should be promptly addressed and managed aggressively. Proper debridement of infected soft tissue, targeted antimicrobial therapy, and achieving soft tissue cover are the main principles of managing graft infections. Negative pressure therapy over temporary soft tissue cover and vascularized muscle flaps is the mainstay of achieving sustained soft tissue cover. This case report describes a patient with an open fracture and a vascular injury complicated with repeated graft trauma, infection, and graft exposure being successfully managed by the use of vascularized muscle flaps. It suggests the potential use of vastus medialis rotational flaps to achieve the soft tissue cover over the vascular exploration sites in the medial aspect of the lower part of the thigh.

## Introduction

Vascular graft infection and graft exposure are considered serious complications following peripheral vascular surgeries. These two complications can cause long-standing morbidity to the patient, which could be both life-threatening and limb-threatening. The reported incidence of vascular graft infections ranges from 0.7% to 10% and it is more common with prosthetic grafts compared to autologous grafts [1]. Microorganisms may reach the vascular graft as a direct spread from the site of the injury, local spread, or through the hematogenous spread [2]. The vascular grafts that are located around the femoral and groin regions are frequently affected [2]. At the same time, peripheral vascular grafts are associated with a higher incidence of infection compared to central vascular grafts [3]. Vascular graft infections are associated with a rate of up to 70% limb loss and increased mortality. Morbidity and mortality are higher in more proximal graft infections [4]. Open procedure, prolonged procedure, Increased body mass index, age, and preexisting vascular disease are some of the risk factors associated with vascular graft infections [5]. Traditionally, these infections were managed with radical surgical debridement followed by extra-anatomical bypasses [3]. However, these procedures are associated with significant perioperative morbidity. As a result, less radical and more conservative approaches were suggested with protracted healing processes. The use of negative pressure wound therapy (NPWT) and vascularized muscle flaps to achieve the soft tissue cover was introduced in the 1980s. Sartorius, rectus femoris, gracilis, rectus abdominis, etc., were suggested as suitable muscle flaps to achieve soft tissue cover over the exposed vascular grafts [6]. Vascularized tissue increases local tissue oxygenation, augments immune cell and antibiotic delivery, and obliterates dead space [7]. The use of vascularized muscle flaps significantly improved the survival and the patency of vascular grafts, thereby reducing the amputation rates and the mortality [8].

We present a patient who presented with a distal femur open fracture with distal superficial femoral vascular injury who was subsequently managed with autologous reverse saphenous interposition vein graft. It was complicated with graft infection and inadequate soft tissue availability to cover the graft, causing graft exposure. He was managed successfully with a vascularized vastus medialis rotational flap followed by split split-thickness skin graft. This article covers the case presentation and an available literature review regarding the use of vascularized muscle flaps to cover exposed, infected, and repeatedly traumatized vascular grafts.

## Case presentation

A 49-year-old man with a history of long-standing diabetes mellitus presented to the emergency room of National Hospital of Sri Lanka following a crush injury with a concrete slab to his right thigh. On admission, his blood pressure was 80/55 mmHg and his pulse rate was 135 beats per minute. A massive transfusion protocol was activated, and the patient was resuscitated with 4 units of red cell concentrates. 

However, following the fall of a heavy object on the thigh, the patient had sustained a Gustilo-Anderson type IIIC right distal femur open fracture. Bleeding from the fracture site was initially managed with compression. The fractured distal segment of the femur has been displaced anteriorly. Patient’s right popliteal artery pulse was not palpable, and the distal limb oxygen saturation was not recordable. However, there was no significant paraesthesia or paresis of the distal limb. But a slight difference in temperature was noted. The suspected ischemic time was 2.5 hours.

Since the patient had multiple hard signs of vascular injury with a single-level fracture, it was decided to proceed with right popliteal artery exploration without imaging. A knee spanning external fixator was implanted by the orthopaedic team in order to provide a stable surgical field for the vascular exploration. The popliteal wound was extended proximally and distally. A complete avulsion of the distal superficial femoral artery (SFA) and superficial femoral vein (SFV) was noted. A single incision four-compartment fasciotomy was performed, and it was noted that all four muscle compartments of the leg were viable. The SFV was ligated, and a distal SFA to popliteal artery reverse saphenous vein graft (RSVG) was done with the greater saphenous vein harvested from the contralateral side. The graft was covered with available soft tissues in the wound. Immediately after the limb perfusion, good graft and distal pulses appeared, and the distal limb saturation was 100% with a satisfactory waveform. The duration of the surgery was 4.5 hours. Postoperatively, the patient was admitted to the intensive care unit.

**Figure 1:**
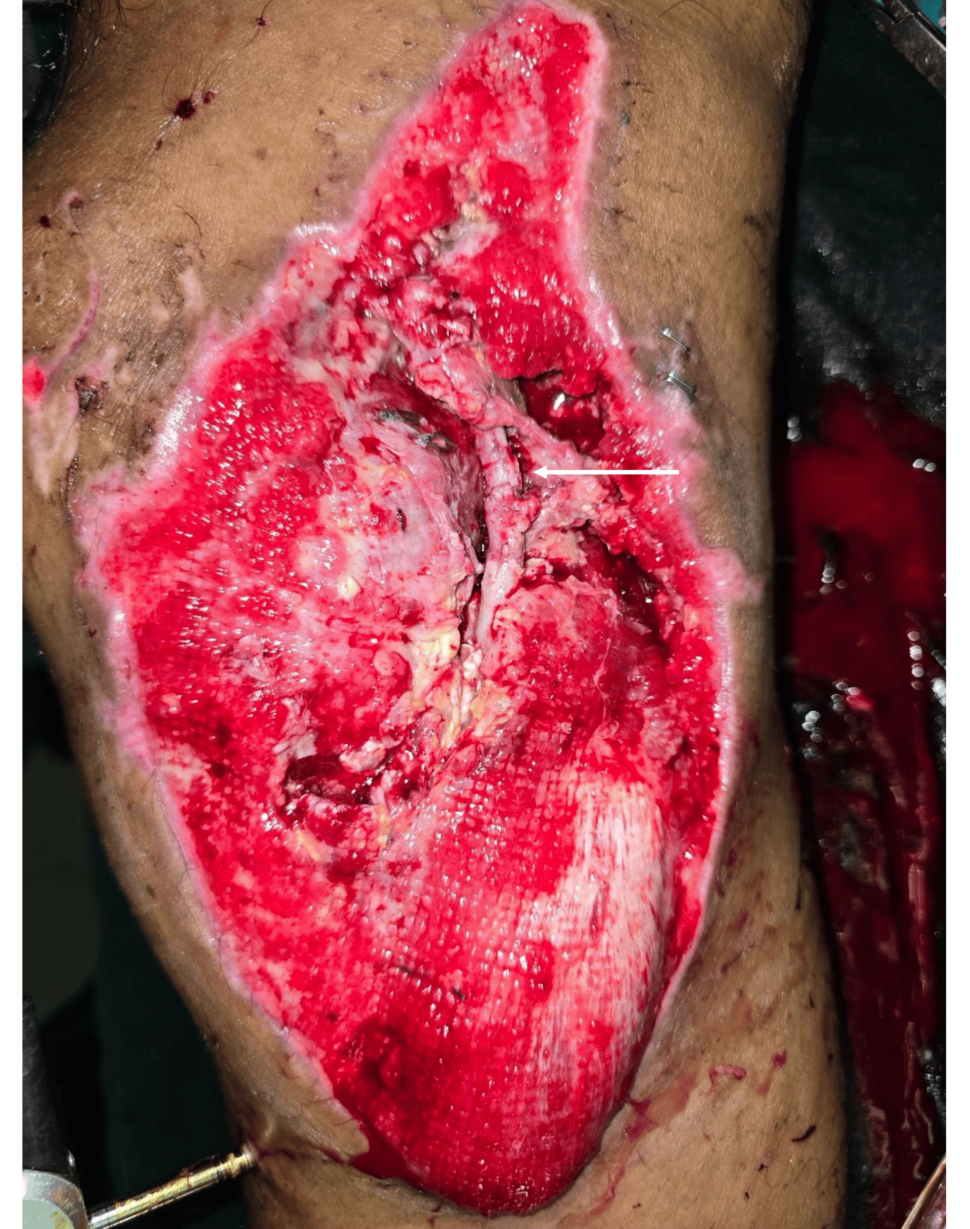
Exposed Segment of the Reverse Saphenous Vein Graft (White Arrow) The white arrow shows the exposed 2-3cm segment of the vascular graft without soft tissue cover. The underlying deep cavities contained necrotic tissues.

The immediate postoperative period of the patient was complicated by rhabdomyolysis, which settled by day 3. However, from day 3 onwards, the patient started developing fever spikes with thrombocytopenia. Blood and urine cultures were negative. The patient was started on intravenous teicoplanin with microbiology opinion. Since abscess formation at the surgical site was a concern, it was decided to proceed with a wound exploration. It was noted that about 3 cm of RSVG is exposed with necrotic tissues in the vicinity (Figure 1).

A wound debridement was performed, and graft cover was achieved with soft tissue approximation. Since the C-reactive protein level of the patient was rising, it was decided to get the assistance of the plastic surgical team to have the definitive wound cover. The patient developed popliteal deep vein thrombosis of the same side on day 6, which could probably have resulted from ligation of the distal SFV. Therefore, he was started on therapeutic anticoagulation. Towards postoperative day 10, the patient gradually improved, and the fever settled. It was decided to proceed with the application of a negative pressure device to the RSVG site and a split-thickness skin graft to the fasciotomy site.

**Figure 2:**
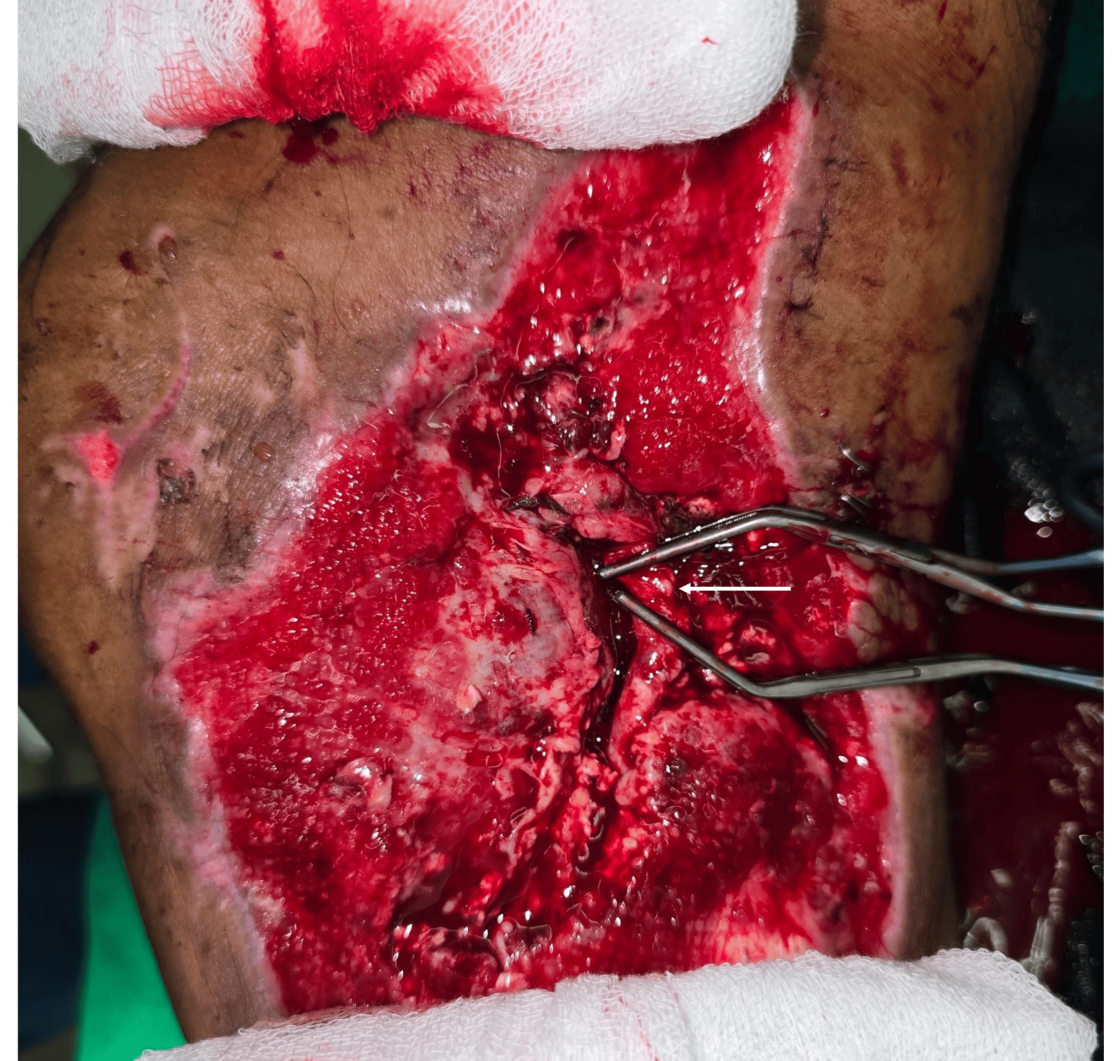
Accidentally Injured Vascular Graft (White Arrow) The white arrow shows the area where an accidental partial thickness laceration (approximately 30% of the circumference) occurred. Two vascular clamps are applied proximal and distal to the site of injury.

However, during the wound debridement for negative pressure device application, the RSVG got an accidental laceration without a segmental loss (Figure 2).

An attempt was made to locate the upper limb and remaining lower limb veins with the view of an extra anatomical repeat reverse vein graft. Unfortunately, none of the veins were suitable for a bypass. Knowing the risk of potential anastomotic breakdown in an infected field leading to catastrophic bleeding and limb loss, it was decided to primarily repair the laceration with the view of an extraanatomical prosthetic graft bypass later.

However, the use of a prosthetic graft even extra-anatomically in an infected surgical field for a vascular anastomosis poses the risk of anastomotic breakdown. Therefore, one more attempt was taken to achieve the soft tissue cover over the RSVG following a careful wound debridement. This time, all the unhealthy tissue was excised. Considering the status of the perivascular graft environment, it was decided to cover the area using a rotational flap with the distal segment of the vastus medialis muscle.

A subfascial dissection was done with sharp and blunt dissection to separate the vastus medialis from the sartorius and adductor magnus, carefully preserving the adductor fascia. It was in the vascular supply for the muscle, which usually enters through the upper one-third, and was left untouched. Once the distal segment of the muscle is adequately mobilized, the flap is rotated over the recipient defect and anchored with absorbable sutures.

**Figure 3:**
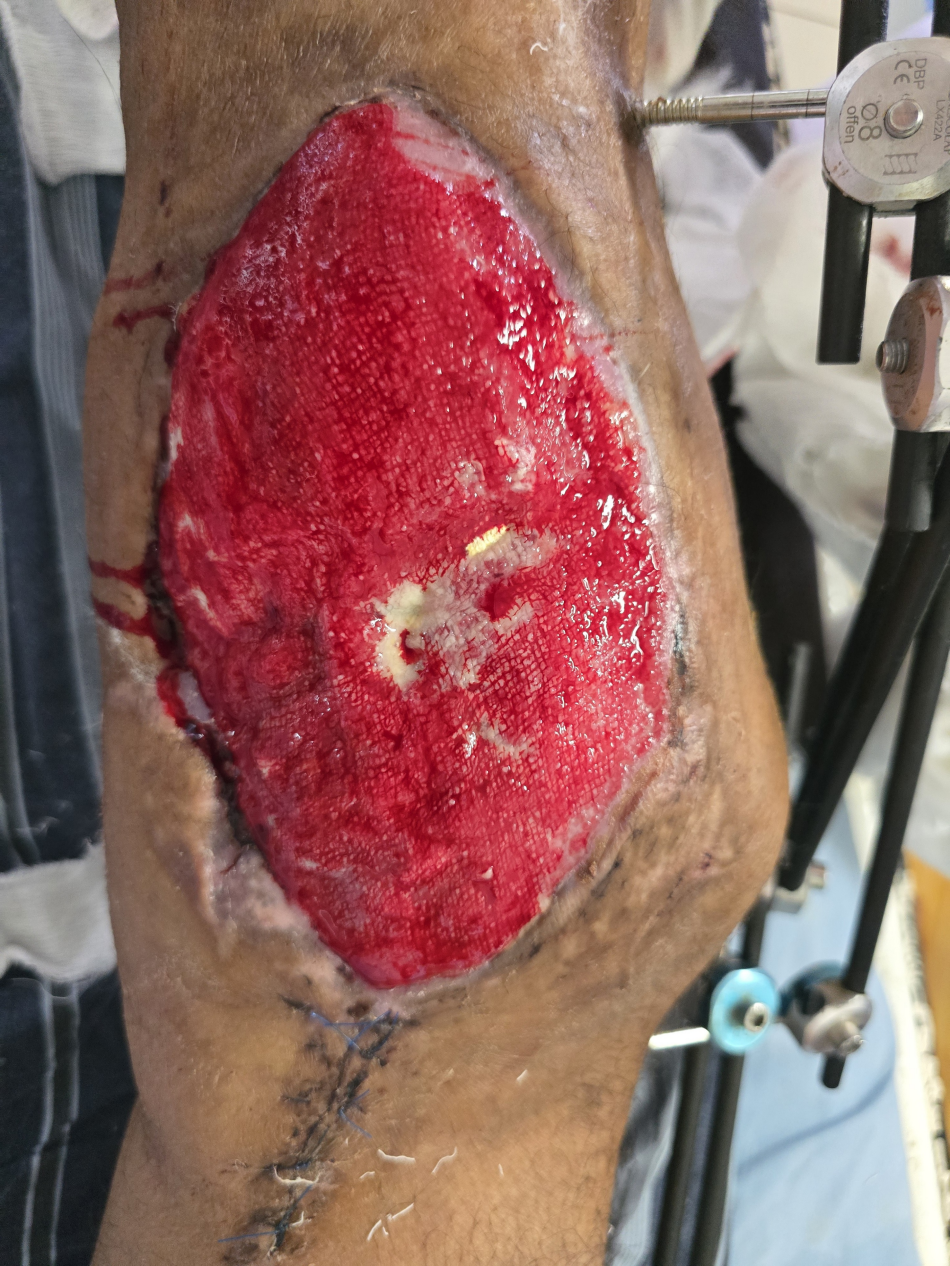
Granulation Over the Vastus Medialis Muscle Flap Compared to Figure 1, all the deep cavities are replaced with healthy granulation tissue and the wound is ready for skin graft

However, with time, granulation tissue appeared over the rotated muscle flap, and the patient underwent a split-thickness skin graft over the granulation tissue (Figure 3).

**Figure 4:**
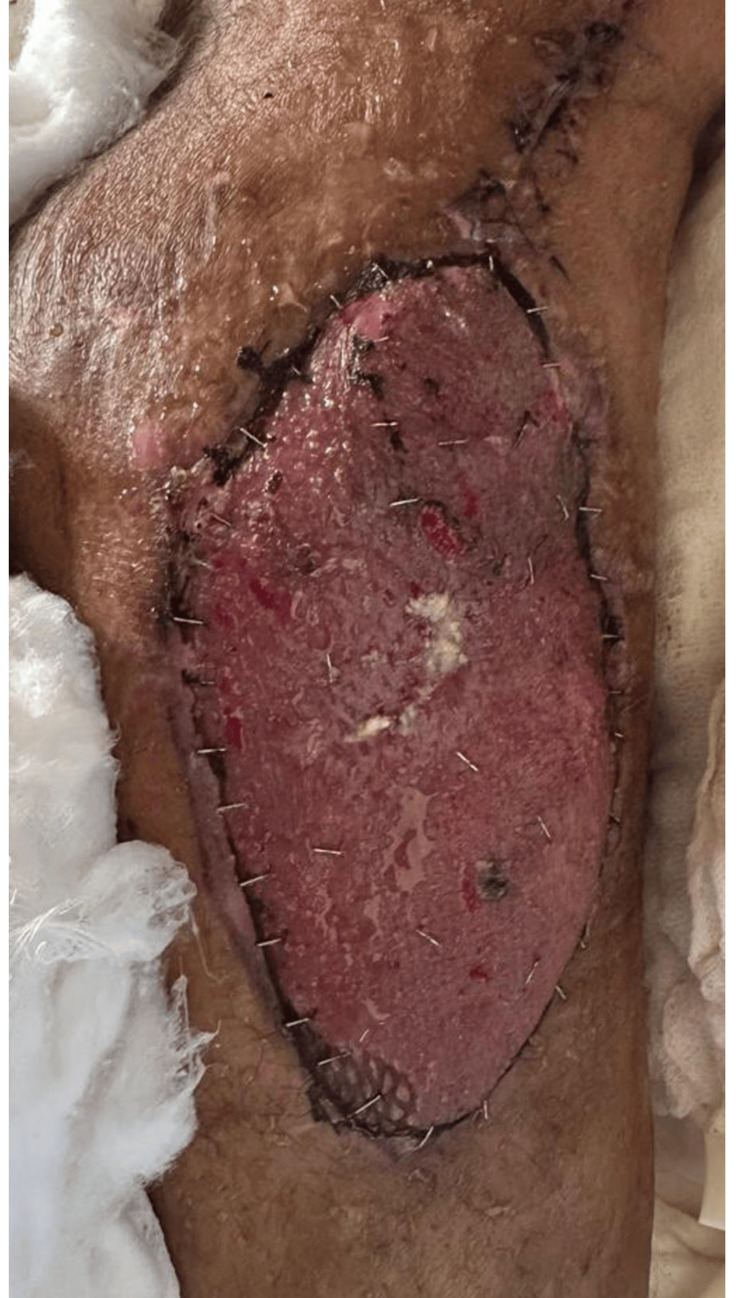
Split Thickness Skin Graft Postoperative Day 5 The appearance of the skin-grafted wound. Please note that more than 80% of the graft is accepted.

 The patient was safely discharged on postoperative day 5 after the split-thickness skin graft (Figure 4).

The patient was taken over by the orthopaedic team for the definitive management of the fracture. The patient was reviewed at the vascular surgical clinic and found to have a viable limb with good distal pulses and adequate soft tissue cover over the vascular anastomosis.

## Discussion

Vascular graft infection is an infection that is established in association with a vascular graft. Graft exposure is having a visible graft at the wound base or wound dehiscence with a tunneling tract to the graft. Graft infections can be divided into early graft infections (within four months) and late graft infections (after four months). Early graft infections are caused by virulent microorganisms, such as S. aureus, E. coli, Pseudomonas, Klebsiella, Proteus, and Enterobacter. Late graft infections are caused by hematogenous spread of organisms or by exposure of vascular grafts to intestinal contents [9]. Virulence of microorganisms is often associated with the production of secreted toxins and enzymes, with a resultant decline in structural integrity of the vessel wall and the release of toxins and enzymes to the perigraft environment and causing graft infection.

The risk factors for vascular graft infections may be divided into patient-related and procedure-related factors. Patient-related factors are age, comorbidities, obesity, and pre-existing infection. Procedure-related factors are emergency surgery, prolonged surgery, surgical sites, and level of contamination. Postoperative risk factors are wound complications, inadequate antimicrobial therapy, and prolonged hospital stay [10].

Szilagyi et al. proposed a classification for graft infections in 1972, depending on the degree of penetration of the infection through different layers of soft tissue. It also had a subclassification depending on the time duration from the time of surgery to the onset of infection [11].

Traditionally, these infections were managed by radical approaches with extra-anatomical bypasses. But these radical approaches were associated with high rates of limb loss, which could be as high as 8-52% [3]. However, as an alternative, Calligaro et al. described managing graft infections with a more conservative approach [6]. 

Using vascularized muscle flaps was introduced recently. These can be used in prophylactic as well as in salvage procedures. Fischer et al. developed the Penn Groin Assessment Scale (PGAS) to identify patients in whom prophylactic muscle flap coverage is beneficial. These authors recommended prophylactic flap coverage to a certain high-risk population with vascular grafts [12]. He also predicted the factors that may necessitate salvage muscle flaps in the future. Prior groin surgery, prosthetic grafts, coronary and peripheral artery disease, and obesity are among them [13]. Evidence shows that performing early muscle flap coverage for exposed, infected vascular grafts improves the rate of vascular graft salvage [7]. In addition, higher salvage rates have been reported following muscular flaps in the setting of acute infection as well [14]. NPWT is also recommended as a safe alternative to muscle flaps. Even though it is not advised to use vacuum-assisted closure directly on vascular grafts as a stand-alone procedure, it has been safely used to achieve healing with secondary intention over bypasses with both autologous and prosthetic grafts [15].

In this patient, factors like emergency surgery, prolonged procedure, underlying diabetes mellitus, high degree of contamination, repeated graft trauma, immediate postoperative haemodynamic stress, and delay in achieving soft tissue cover could have contributed to graft infection. Although autologous graft infections are rare compared to prosthetic grafts, the tissue culture taken from the immediate vicinity of the graft was positive for Acinetobacter, which is a pathogen that is not known to cause frequent graft infections. This could have been due to the direct spread of the organisms from the site where the injury took place or acquired during the intensive care unit stay. Ongoing rhabdomyolysis, positive tissue cultures, and being on therapeutic anticoagulation would have contributed to the delay in achieving soft tissue cover. Although venous reconstruction is recommended above the trifurcation level in traumatic vascular injury, due to the gross contamination of the surgical field, it was decided to ligate the SFV. This obviously led to deep vein thrombosis, even though immobilization might have contributed. When the graft got accidentally injured, the better and safer option would have been to do a re-SVG bypass. But due to the unavailability of the autologous conduits, the team was left without an option and had to take the risk of doing a direct vascular repair in an infected field. We assume that the relatively young age, good nutritional status, and satisfactory physical health contributed to the overall positive outcome. Even though NPWT was considered as an option at one point, it was abandoned since using vacuum-assisted closure directly over the anastomosis does not have favorable outcomes. Ultimately use of the vascularized muscle flap led to better wound healing and a patent graft with a viable limb.

## Conclusions

Vascular graft infections and graft exposure are nightmares for vascular surgeons. These two complications will lead to a poor quality of life with limb and life loss. Therefore, graft infections and exposures should be promptly addressed. Proper debridement, appropriate antimicrobial therapy, and use of NPWT or vascularized muscle flaps are ways for graft salvage. Specifically from this case report, we would like to bring about the fact that using the distal insertion end of the vastus medialis as a rotational flap to cover vascular exploration sites in the medial aspect of the knee is an option for similar scenarios.
